# Longitudinal Volumetric Assessment of Ventricular Enlargement in Pet Dogs Trained for Functional Magnetic Resonance Imaging (fMRI) Studies

**DOI:** 10.3390/vetsci7030127

**Published:** 2020-09-04

**Authors:** Eva Gunde, Kálmán Czeibert, Anna Gábor, Dóra Szabó, Anna Kis, Attila Arany-Tóth, Attila Andics, Márta Gácsi, Enikő Kubinyi

**Affiliations:** 1 Department and Clinic of Surgery and Ophthalmology, University of Veterinary Medicine, 1078 Budapest, Hungary; egunde@dal.ca (E.G.); aaranytoth@yahoo.com (A.A.-T.); 2 Department of Ethology, Institute of Biology, ELTE Eötvös Loránd University, 1117 Budapest, Hungary; annagabor33@gmail.com (A.G.); szaboodoora@gmail.com (D.S.); attila.andics@gmail.com (A.A.); marta.gacsi@gmail.com (M.G.); kubinyie@gmail.com (E.K.); 3 MTA-ELTE (Hungarian Academy of Sciences–Eötvös Loránd University) ‘Lendūlet Neuroethology of Communication Research Group, 1117 Budapest, Hungary; 4 Psychobiology Research Group, Institute of Cognitive Neuroscience and Psychology, Research Centre for Natural Sciences, 1117 Budapest, Hungary; vargane.kis.anna@ttk.mta.hu; 5 MTA-ELTE Comparative Ethology Research Group, 1117 Budapest, Hungary

**Keywords:** awake canine neuroimaging, ventriculomegaly, hydrocephalus, attention, brain, MRI

## Abstract

Background: Recent studies suggest that clinically sound ventriculomegaly in dogs could be a preliminary form of the clinically significant hydrocephalus. We evaluated changes of ventricular volumes in awake functional magnetic resonance imaging (fMRI) trained dogs with indirectly assessed cognitive abilities over time (thus avoiding the use of anaesthetics, which can alter the pressure). Our research question was whether ventricular enlargement developing over time would have any detrimental effect on staying still while being scanned; which can be extrapolated to the ability to pay attention and to exert inhibition. Methods: Seven healthy dogs, 2–8 years old at the baseline scan and 4 years older at rescan, participated in a rigorous and gradual training for staying motionless (<2 mm) in the magnetic resonance (MR) scanner without any sedation during 6 minute-long structural MR sequences. On T1 structural images, volumetric analyses of the lateral ventricles were completed by software guided semi-automated tissue-type segmentations performed with FMRIB Software Library (FSL, Analysis Group, Oxford, UK). Results and conclusion: We report significant enlargement for both ventricles (left: 47.46 %, right: 46.07 %) over time while dogs retained high levels of attention and inhibition. The results suggest that even considerable ventricular enlargement arising during normal aging does not necessarily reflect observable pathological changes in behavior.

## 1. Introduction 

Hydrocephalus is an abnormal accumulation of cerebrospinal fluid (CSF) that requires clinical attention and may result in a diversity of neurological symptoms, including visual or auditory impairment, seizures, incoordination, abnormal behavior such as depression, hyper-excitability, and cognitive dysfunction [1,2,3,4].

Diagnosis of hydrocephalus in dogs is based on the assessment of the clinical presentation and diagnostic imaging results, such as magnetic resonance imaging (MRI). On the MR image, internal hydrocephalus can present with severe or moderate dilation of ventricles, mostly visible at the lateral ventricles, and often with a well-observable hemispheric asymmetry [1,5,6]. Despite its characteristic appearance on the MRI, however, diagnosis of internal hydrocephalus bares some challenges, as it shares similar imaging features with a clinically silent ventriculomegaly [1,3,4,7,8]. It is important to highlight that currently, there is still no consensus and no clearly defined delineation between these two conditions. This ambiguity can make arriving to the diagnosis and clinical decisions difficult. Based on the literature, ventricular enlargement is not always associated with clinical signs of hydrocephalus or increased intracranial pressure. Enlarged ventricles have been described as a frequent normal morphologic variation in brachycephalic dogs [3,5,9,10] and in Labrador retrievers [7], as well being associated with normal aging [11,12,13]. Further, Thomas argued that any condition causing thinning of the brain parenchyma, which ultimately leaves a vacant space to be filled by CSF, should not be regarded as hydrocephalus [8].

In contrast, a recent study suggested that enlargement of the lateral ventricles seen in brachycephalic dogs might be a consequence of periventricular loss of white matter tissue, due to moderately or intermittently increased intracranial pressure provoking a temporary ischemic effect and ultimately white matter loss [14]. The same research group also noted a reduced periventricular cerebral blood perfusion in clinically sound dogs [15], shown to be decreased in humans with normal pressure hydrocephalus as well [16,17]. They proposed that canine ventriculomegaly is not a physiological variant of ventricular morphology as previously reported, but possibly a preliminary or arrested form of internal hydrocephalus [14,15]. In ventriculomegaly, the accumulation of CSF and distention of the ventricles may occur very slowly, which allows the brain to adapt to pathological changes, such as periventricular parenchymal thinning and decreased perfusion. Further, to notice the detrimental effects of ventriculomegaly in dogs, a long term accumulation of ischemic insults may be necessary [4].

It has been demonstrated that hydrocephalus in humans presents with progressive cognitive decline and ultimately dementia [18]. As dogs appear to be an ideal model of hydrocephalus [1,19,20] and of aging-related disorders [12,21,22,23,24,25,26,27], it is reasonable to predict that ventriculomegaly present in dogs would result in cognitive or neurological decline over time as well.

To verify these postulations, follow-up studies examining ventricular volume and cognitive performance are needed. To our knowledge, only a few follow-up MRI studies assessed ventricular volume changes over time [12,24,28]. Two of these studies investigated ventricular enlargement only for short time periods and in very young dogs (<90 weeks). Additionally, the animals of these studies underwent drug therapies not relevant to hydrocephalus in both of these studies [24,28]. The third study examined healthy dogs between 8–11 years throughout 3 years and found progressive ventricular enlargement with aging [12]. The cognitive abilities of the participating animals however were not considered.

One of the challenges of MR imaging, particularly functional MRI (fMRI), is motion susceptibility. Motion during the scanning time is measured in three directions and it cannot exceed a strict limit (e.g., 2 mm in our previous studies) in order to acquire adequate image quality for functional and structural scans. Fulfilling this task, which is staying completely motionless in the MR machine without any sedation, necessitates a variety of cognitive skills, such as sustained attention (e.g., staying awake) and inhibition (e.g., staying motionless up to 8 minutes regardless of the presented stimuli). Both attention and inhibition are attributed to frontal lobe activity, and frontal lobe deficits have been shown to be the earliest signs of hydrocephalus in humans [18]. In the present study we assessed ventricular volume changes over a four-year period in fMRI trained dogs, that retained their ability to stay motionless within the strict limits of our protocol, and thus presumably maintained a good status of their frontal lobe functionality.

## 2. Materials and Methods

### 2.1. Aim of the Study

Our aim was to investigate whether lateral ventricle enlargement would be observable based on MR measurements acquired from healthy dogs with a four-year-long lapse. Given that four years take up a significant percentage of the canine lifespan, we predicted that such brain anatomy changes would be observable in this longitudinal setting. Since all dogs included in the study retained their ability to stay motionless during the MRI scan, we assumed that any observable ventricular enlargement would not necessarily be associated with pathological changes in cognitive performance.

### 2.2. Subjects

**Figure 1:**
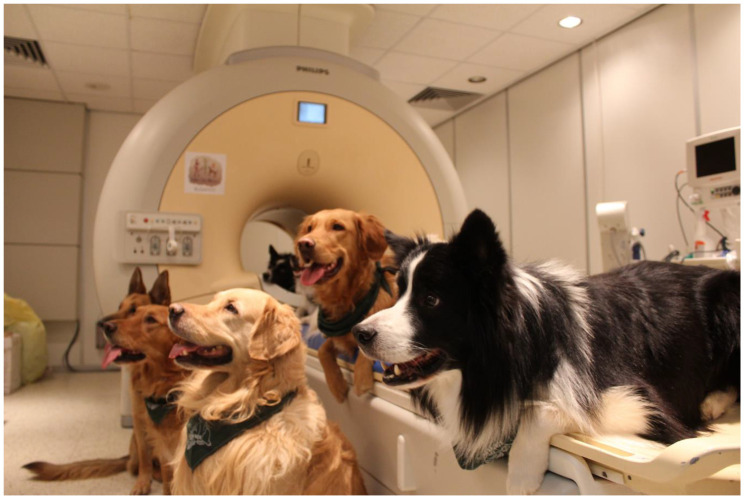
Participants of the Senior Family Project, fMRI trained dogs.

Our subjects were fMRI trained dogs (three golden retrievers, and four border collies; four males, three females) that underwent repeated structural and functional MRI examination and satisfied the criteria of having at least two scans four years apart to secure a measurable change in volume and being at least 6 years of age at the second scan. The latter criterion was based on previous literature showing increased ventricular dilation after 6 years of age [11]. The average age at the baseline scanning was 46.3 ± 26 months with a range of 23–60 months, while the average age at the second scan was 96 ± 24 months with a range of 76–144 months. All animals were companion dogs (Figure 1) whose owners volunteered to participate in the project and had been trained using methods fully compatible with positive animal welfare following the national and European guidelines for animal care. Experimental procedures were approved by the local ethical committee of organization of food safety and animal health agency (Hungarian Directorate for Food Chain Safety and Animal Health XIV-I-001/520-4/2012, Budapest, Hungary). All subjects were under private veterinary care with no reported physiological or neurological disorder and vaccinated according to regulations.

### 2.3. Training of the Dogs

**Figure 2:**
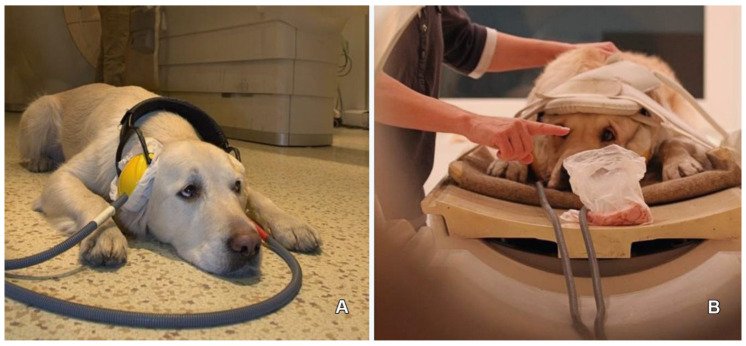
Training of the dogs. During training, getting used to the headphones (**A**), and in the MR scanner, learning to keep eye contact while lying motionless with headphones and the receiver coil secured (**B**).

Description of the training is published elsewhere in detail [29,30]. In short, dogs underwent rigorous gradual training using positive reinforcement and social learning for at least 6 months. Using a mock scanner, they have learnt to climb on a ramp and to lie on the table motionless for gradually increasing time, and got used to the headphones (Figure 2A). After acquiring these skills, the dogs were trained in the actual scanner, were habituated to MR noise, got accustomed to the moving table, and to the receiver coil placed on their heads (Figure 2B). Besides positive reinforcement in the form of food and praise, social learning was also applied, which involved novice dogs observing a trained dog performing the task and being rewarded (model-rival training). On average, the animals needed 12 training sessions in the mock scanner and 7 in the MR machine before they could satisfy the criteria of staying motionless (no more than 2 mm movement) during the 6 minute scanning session. During the scanning, the dogs were not restrained and could leave the MR suite at any time. The training was performed by the staff of the Family Dog Project at the Department of Ethology of Eötvös Loránd University.

### 2.4. Imaging Technique and Analyses

The data were collected using a 3 Tesla Philips Achieva scanner (Philips Medical Systems, The Netherlands). As a surface coil, Philips SENSE Flex-M (Koninklijke Philips N.V., Amsterdam, the Netherlands), a two circular element system was used; one was placed on the scanner table, while the other was secured on the animal’s head. For the structural image Turbo Field Echo sequence was used with the following parameters; repetition time (TR) = 9.9 s, time echo (TE) = 4.6 s; flip angle = 8°; field of view (FOV) = 255 × 255, slice number = 109 with 1 × 1 × 1 mm^3^ isovoxel spatial resolution, and no gap.

During the scanning, the dogs’ handlers were in the scanner room to provide comfort. Motion was measured on three planes: transverse, sagittal, and dorsal. The animals included in the study did not move more than 2 mm in any direction.

**Figure 3:**
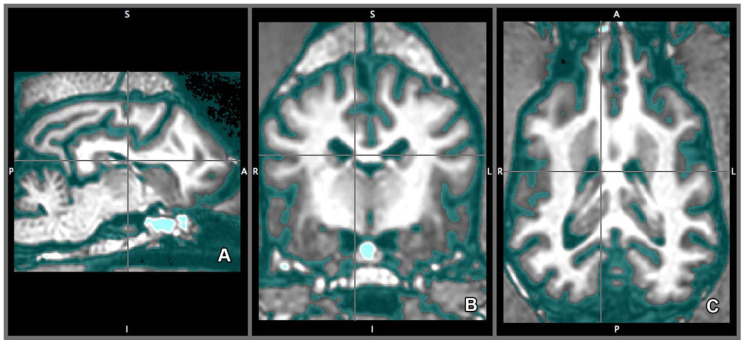
Cerebrospinal fluid (CSF) template of the brain. CSF segmentation on the sagittal (**A**), transverse (**B**), and on the dorsal plane (**C**). On CSF template of the software, the area highlighted in blue signifies the CSF or any other tissue that has the same intensity value. That includes the lateral ventricles. Directions are referred as superior (S), inferior (I), posterior (P), anterior (A), left (L) and right (R).

Volumetric analysis of the right and left lateral ventricles were completed by software-guided semi-automated tissue-type segmentation [31,32] using 0.333 mm off-line isovoxel resolution. FAST (Functional Magnetic Resonance Imaging of the Brain’s (FMRIB) Automated Segmentation Tool) segments MR image of the brain into different tissue types (gray matter, white matter, CSF) based on the intensity of a given voxel, and creates probability models that help to determine tissue type around the borders. The program uses bias field-correction for removing intensity variations across space that may be present due to inhomogeneity of the radio-frequency field, applies spatial neighborhood information, creates partial volume model, and based on the information, iterates the tissue type segmentation. FAST is robust, reliable, and not sensitive to noise [33] (Figure 3).

**Figure 4:**
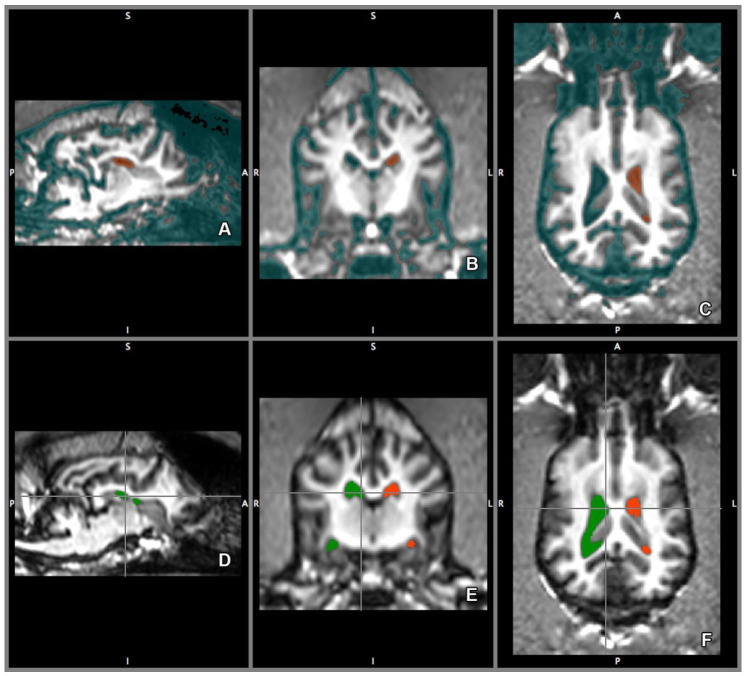
Lateral ventricle template. Based on the CSF segmentation template, the left ventricle is delineated (red area, **A**–**C**), and left (red area) and right (green area) ventricles delineated (**D**–**F**) using 3-dimensional tracing. The lateral ventricles are traced separately, creating a template for each of them, the volume of the templates then were calculated. Directions are referred as superior (S), inferior (I), posterior (P), anterior (A), left (L) and right (R).

Based on the CSF segmentation templates, the lateral ventricles were manually delineated using all three planes. The transverse plane was chosen as the primary direction (Figure 4B,E). The delineated ventricles then were measured by counting the number of voxels (three-dimensional pixels).

### 2.5. Morphology and Size of the Measured Brains

We eliminated false ventricular size differences due to brain shape and size differences by choosing specific breeds for the study. Based on an ongoing canine brain bank project, the participating dogs, golden retrievers, and border collies, had very similar brains in size and shape [34].

### 2.6. Statistical Analyses

Statistical analyses were completed using SPSSv25 (IBM software package, https://www.ibm.com/products/spss-statistics). Changes in ventricular volumes over time were calculated using the Friedman test. The Friedman test was chosen because of the mandatory criteria of parametric statistics, including normal distribution of the data and homogeneous variance, were not met possibly due to the small sample size. Accordingly, the result is better represented by the median than the mean. The Friedman test is the non-parametric alternative of one-way analysis of variance (ANOVA) with repeated measures, in which one group is measured two or more times.

## 3. Results

**Figure 5:**
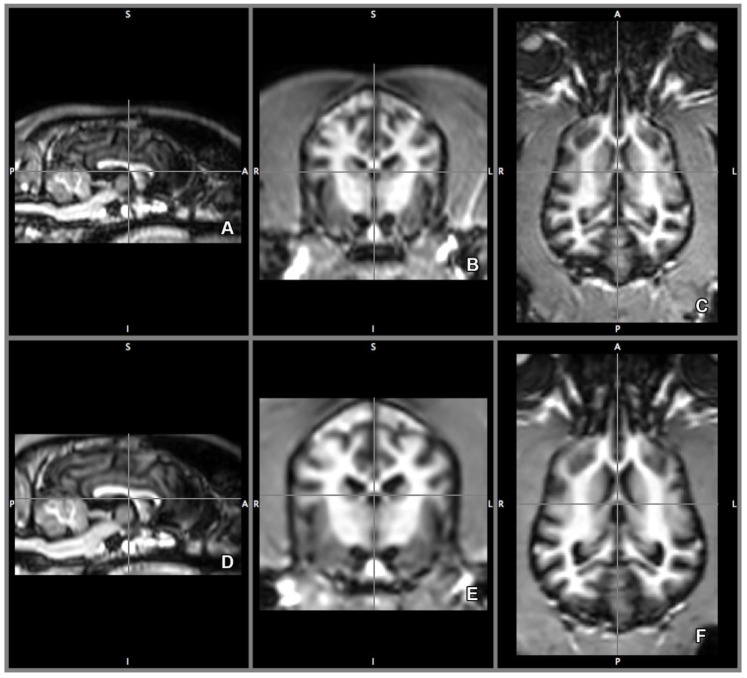
Ventricular changes over time of the same dog, at the 1st scan (**A**–**C**) and 4 years later at the 2nd scan (**D**–**F**). Directions are referred as superior (S), inferior (I), posterior (P), anterior (A), left (L) and right (R).

**Figure 6:**
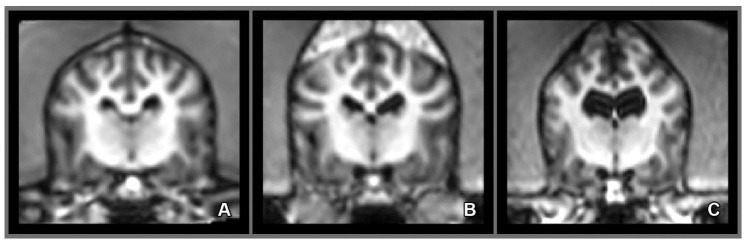
Baseline magnetic resonance (MR) images of three tested dogs. Mildly (**A**), moderately (**B**) and extremely enlarged (**C**) lateral ventricles are presented.

A statistically significant difference between the baseline and rescan period was demonstrated for both the left and the right sides (χ^2^ = 7.000 *p* = 0.008 each, Figure 5). One of the dogs had significantly larger baseline and rescan lateral ventricles than the rest of the animals, but when this animal was excluded, the difference remained still significant for both sides χ^2^ = 6.000 *p* = 0.014. Individual enlargement of the lateral ventricles calculated in percentage revealed comparable overall enlargement for both; left = 47.46 % and right = 46.07% sides. The results of the individual enlargements are presented in Table 1. Finally, there were considerable individual variations regarding the size of the lateral ventricles (Figure 6).

## 4. Discussion

In line with our hypotheses, lateral ventricle enlargement was measurable and significant at the four-year lapse mark on both sides. Unfortunately, our sample size was small (N = 7) and therefore nonparametric statistics were the only option, which may have eroded some information, such as the asymmetry of the enlargement of the two hemispheres (Table 1). When, however, expressed in average percentage, it was 47.46% for the left and 46.07% for the right side for the seven dogs, which shows approximately similar vulnerability for both sides with marked individual differences. A previous study noted a significant widening of the lateral ventricles over a 3 year period [12]. We chose a 4 year mark to most possibly secure a measurable change in volume. The relatively old age of the animals, particularly at the rescan (76 months), was also essential, as the genuine volume change should have taken off only after 6 years of age (72 months) [11].

The work by Su and colleagues [12] is the only similar longitudinal study measuring ventricular enlargement over the years. They reported a lot smaller (4.5%) lateral ventricle enlargement in 8–11 year old dogs compared to our results; however, the methods of the two studies are not comparable. The first important difference is that we expressed our enlargement in its actual volume, while in their study, ventricular enlargement was reported as a lateral ventricle/cerebrum ratio. The second important difference is the resolution of the MR images. While the previous study had 1.2–1.5 mm slice thickness, our on-line slice thickness was 1 mm while our off-line was 0.33 mm, meaning that we had much higher resolution enabling superior accuracy.

Although our sample size was small and we could not control for the effect of age in the statistical analysis, individual variations in the magnitude of ventricular enlargement are still striking. It is improbable that these individual variations are related to differences in brain size and shape as all dogs included in the study, golden retrievers and border collies, were similar in these characteristics. While ventricular enlargement seems to follow a predictable fashion as a function of age [11] our sample suggests that individual variations diverging from the predictable age-related widening may not be uncommon. For example, the oldest dog (99 months at baseline–144 months at rescan) had the smallest enlargement. The extent of individual variations of the widening may be an important consideration in clinical settings and reminds us of the need for additional behavioral assessments apart from the currently exercised imaging procedures. Due to the applied fMRI training method (described in detail by Andics et al. [29]), which is based on a positive reinforcement techniques (praises and food rewards) and social learning (model-rival training), we ensured that the dogs are motivated to cooperate and please the owner and the trainer (see the video abstract of the article [29], with the information about the training from 2:00).

Even though the tested animals cannot be categorized as hydrocephalic dogs, the substantial ventricular enlargement seen in six out of the seven animals may suggest that these animals would present some neurocognitive deficit if ventriculomegaly was truly a preliminary form of hydrocephalus. Neurocognitive implications of hydrocephalus or ventriculomegaly in dogs are still under investigation, but in humans, several cognitive symptoms, including attention and inhibition insufficiencies, have been confirmed to be hallmarks of hydrocephalus [18]. The animals in our study were not directly tested for such capacities, but their ability to stay in the MR scanner with minimal motion (<2 mm) during scan and rescan strongly suggests a high functioning attention and inhibition system. Based on the current result, the notion of retaining high functioning in cognitive tasks despite substantial ventricular enlargement is also important in a point of view of clinical decisions and therapy, particularly when only neuroimaging data are available.

Apart from neuroimaging, to our knowledge, the other currently available clinical procedure when ventriculomegaly or hydrocephalus is suspected is neurological testing. Although these clinical assessments are invaluable, they can only measure crude behavioral or neurological impairments, such as gait abnormalities, house soiling, disorientation, sleep-wake cycle disturbances, and interaction changes [35], reflecting significant irreversible brain tissue damage already being present at that point. In order to detect the commencement of age-related cognitive decline, the covert manifestation of these disturbances or the lack of them, more sensitive neurocognitive measures tapping on early signs of hydrocephalus or ventriculomegaly are needed. We attempted to indirectly assess certain cognitive skills, such as attention and inhibition, and only included dogs that seemed to be intact regarding these skills based on their motion parameters while being scanned. In the future, further studies are needed to directly assess all cognitive functions that can be affected in ventriculomegaly, and to test how they are related to anatomical MR data.

This study is the first to measure the volume of the lateral ventricles in their normal anatomical position in conscious dogs. Although volumetric measurements could be more precise with long scanning sessions (e.g., 20–30 minute sequences), due to gaining better contrast and higher resolution on MR images, these types of imaging however necessitate the anesthesia of the animal. Anesthetics, such as propofol and isoflurane, but also the sheer position of the sleeping animal have intracranial pressure modifying properties [36,37,38] and may influence the volumetric outcome of the ventricles.

In conclusion, this study testifies significant individual variations in ventricle size and variability in the extent of the enlargement. Further, our study points out that even with considerable ventriculomegaly, the neurocognitive function can stay intact. This is the first study that measures ventricle volume changes over time in conscious dogs without the potential altering effect of anesthesia. These results hold valuable information in clinical settings when arriving to proper diagnosis and treatment plan based on neuroimaging data. Finally, the current study highlights the need for the development of sensitive neurocognitive measures that can be also applied in animal clinics.

## Figures and Tables

**Table 1:** Ventricle volumes (left and right) of the seven dogs presented individually and in sum. The enlargement is expressed in percentage comparing volume measured at the 1st and the 2nd scan.

Ventricular Enlargement between 1st and 2nd Measurement of the 7 Dogs				
	Age in months at the time of the scan		Enlargement in %	
ID	1st	2nd	left	Right
1	39	82	61.96	24.04
2	27	78	48.32	86.10
3	60	115	44.21	46.60
4	99	144	6.45	11.33
5	44	95	70.77	0.34
6	23	76	41.52	56.15
7	32	80	58.98	97.92
Average			47.46	46.07

## References

[1] Vullo T., Korenman E., Manzo R.P., Gomez D.G., Deck M.D.F., Cahill P.T. (1997). Diagnosis of cerebral ventriculomegaly in normal adult beagles using quantitative MRI. Vet. Radiol. Ultrasound.

[2] Thomas W.B. (1999). Nonneoplastic disorders of the brain. Clin. Tech. Small Anim. Pract..

[3] Ryan C.T., Glass E.N., Seiler G., Zwingenberger A.L., Mai W. (2014). Magnetic resonance imaging findings associated with lateral cerebral ventriculomegaly in English bulldogs. Vet. Radiol. Ultrasound.

[4] Laubner S., Ondreka N., Failing K., Kramer M., Schmidt M.J. (2015). Magnetic resonance imaging signs of high intraventricular pressure-comparison of findings in dogs with clinically relevant internal hydrocephalus and asymptomatic dogs with ventriculomegaly. BMC Vet. Res..

[5] Kii S., Uzuka Y., Taura Y., Nakaichi M., Takeuchi A., Inokuma H., Onishi T. (1997). Magnetic resonance imaging of the lateral ventricles in beagle-type dogs. Vet. Radiol. Ultrasound.

[6] Esteve-Ratsch B., Kneissl S., Gabler C. (2001). Comparative evaluation of the ventricles in the Yorkshire terrier and the German shepherd dog using low-field MRI. Vet. Radiol. Ultrasound.

[7] Haan C.E.D., Kraft S.L., Gavin P.R., Wendling L.R., Griebenow M.L. (1994). Normal variation in size of the lateral ventricles of the labrador retriever dog as assessed by magnetic resonance imaging. Vet. Radiol. Ultrasound.

[8] Thomas W.B. (2010). Hydrocephalus in dogs and cats. Vet. Clin. Small Anim. Pract..

[9] Vite C.H., Insko E.K., Schotland H.M., Panckeri K., Hendricks J.C. (1997). Quantification of cerebral ventricular volume in English bulldogs. Vet. Radiol. Ultrasound.

[10] Driver C.J., Chandler K., Walmsley G., Shihab N., Volk H.A. (2013). The association between Chiari-like malformation, ventriculomegaly and seizures in cavalier King Charles spaniels. Vet. J..

[11] Su M., Head E., Brooks W.M., Wang Z., Muggenburg B.A., Adam G.E., Sutherland R., Cotman C.W., Nalcioglu O. (1998). Magnetic resonance imaging of anatomic and vascular characteristics in a canine model of human aging. Neurobiol. Aging.

[12] Su M.-Y., Tapp P.D., Vu L., Chen Y.-F., Chu Y., Muggenburg B., Chiou J.-Y., Chen C., Wang J., Bracco C. (2005). A longitudinal study of brain morphometrics using serial magnetic resonance imaging analysis in a canine model of aging. Prog. Neuropsychopharmacol. Biol. Psychiatry.

[13] Head E. (2011). Neurobiology of the aging dog. AGE.

[14] Schmidt M.J., Laubner S., Kolecka M., Failing K., Moritz A., Kramer M., Ondreka N. (2015). Comparison of the relationship between cerebral white matter and grey matter in normal dogs and dogs with lateral ventricular enlargement. PLoS ONE.

[15] Schmidt M.J., Kolecka M., Kirberger R., Hartmann A. (2017). Dynamic susceptibility contrast perfusion magnetic resonance imaging demonstrates reduced periventricular cerebral blood flow in dogs with ventriculomegaly. Front. Vet. Sci..

[16] Momjian S., Czosnyka Z., Czosnyka M., Pickard J.D. (2004). Link between vasogenic waves of intracranial pressure and cerebrospinal fluid outflow resistance in normal pressure hydrocephalus. Br. J. Neurosurg..

[17] Jeppsson A., Zetterberg H., Blennow K., Wikkelsø C. (2013). Idiopathic normal-pressure hydrocephalus: Pathophysiology and diagnosis by CSF biomarkers. Neurology.

[18] Iddon J.L., Pickard J.D., Cross J.J.L., Griffiths P.D., Czosnyka M., Sahakian B.J. (1999). Specific patterns of cognitive impairment in patients with idiopathic normal pressure hydrocephalus and Alzheimer’s disease: A pilot study. J. Neurol. Neurosurg. Psychiatry.

[19] Yamada H., Yokota A., Haratake J., Horie A. (1996). Morphological study of experimental syringomyelia with kaolin-induced hydrocephalus in a canine model. J. Neurosurg..

[20] Vullo T., Manzo R., Gomez D.G., Deck M.D., Cahill P.T. (1998). A canine model of acute hydrocephalus with MR correlation. Am. J. Neuroradiol..

[21] Tapp P.D., Siwak C.T., Gao F.Q., Chiou J.-Y., Black S.E., Head E., Muggenburg B.A., Cotman C.W., Milgram N.W., Su M.-Y. (2004). Frontal lobe volume, function, and β-amyloid pathology in a canine model of aging. J. Neurosci..

[22] Tapp P.D., Chu Y., Araujo J.A., Chiou J.-Y., Head E., Milgram N.W., Su M.-Y. (2005). Effects of scopolamine challenge on regional cerebral blood volume. A pharmacological model to validate the use of contrast enhanced magnetic resonance imaging to assess cerebral blood volume in a canine model of aging. Prog. Neuropsychopharmacol. Biol. Psychiatry.

[23] Head E. (2013). A canine model of human aging and Alzheimer’s disease. Biochim. Biophys. Acta.

[24] Katz M.L., Coates J.R., Sibigtroth C.M., Taylor J.D., Carpentier M., Young W.M., Wininger F.A., Kennedy D., Vuillemenot B.R., O’Neill C.A. (2014). Enzyme replacement therapy attenuates disease progression in a canine model of late-infantile neuronal ceroid lipofuscinosis (CLN2 disease). J. Neurosci. Res..

[25] Schütt T., Helboe L., Pedersen L.Ø., Waldemar G., Berendt M., Pedersen J.T. (2016). Dogs with cognitive dysfunction as a spontaneous model for early Alzheimer’s disease: A translational study of neuropathological and inflammatory markers. J. Alzheimers Dis..

[26] Mazzatenta A., Carluccio A., Robbe D., Giulio C.D., Cellerino A. (2017). The companion dog as a unique translational model for aging. Semin. Cell Dev. Biol..

[27] Sándor S., Kubinyi E. (2019). Genetic pathways of aging and their relevance in the dog as a natural model of human aging. Front. Genet..

[28] Hines C.D.G., Song X., Kuruvilla S., Farris G., Markgraf C.G. (2015). Magnetic resonance imaging assessment of the ventricular system in the brains of adult and juvenile beagle dogs treated with posaconazole IV Solution. J. Pharmacol. Toxicol. Methods.

[29] Andics A., Gácsi M., Faragó T., Kis A., Miklósi A. (2014). Voice-sensitive regions in the dog and human brain are revealed by comparative fMRI. Curr. Biol. CB.

[30] Andics A., Gábor A., Gácsi M., Faragó T., Szabó D., Miklósi Á. (2016). Neural mechanisms for lexical processing in dogs. Science.

[31] Smith S.M., Jenkinson M., Woolrich M.W., Beckmann C.F., Behrens T.E.J., Johansen-Berg H., Bannister P.R., De Luca M., Drobnjak I., Flitney D.E. (2004). Advances in functional and structural MR image analysis and implementation as FSL. NeuroImage.

[32] Jenkinson M., Beckmann C.F., Behrens T.E.J., Woolrich M.W., Smith S.M. (2012). FSL. NeuroImage.

[33] Zhang Y., Brady M., Smith S. (2001). Segmentation of brain MR images through a hidden Markov random field model and the expectation-maximization algorithm. IEEE Trans. Med. Imaging.

[34] Czakó L. (2018). Egy új hazai kezdeményezés: Kutya Agy- és Szövetbank Létesítése és Működtetése [A New Hungarian Initiative: Creating the Canine Brain and Tissue Bank]. Master’s thesis.

[35] Madari A., Farbakova J., Katina S., Smolek T., Novak P., Weissova T., Novak M., Zilka N. (2015). Assessment of severity and progression of canine cognitive dysfunction syndrome using the CAnine DEmentia Scale (CADES). Appl. Anim. Behav. Sci..

[36] Yamada S., Ducker T.B., Perot P.L. (1975). Dynamic changes of cerebrospinal fluid in upright and recumbent shunted experimental animals. Pediatr. Neurosurg..

[37] Ravussin P., Guinard J.P., Ralley F., Thorin D. (1988). Effect of propofol on cerebrospinal fluid pressure and cerebral perfusion pressure in patients undergoing craniotomy. Anaesthesia.

[38] Harrington M.L., Bagley R.S., Moore M.P., Tyler J.W. (1996). Effect of craniectomy, durotomy, and wound closure on intracranial pressure in healthy cats. Am. J. Vet. Res..

